# Light-emitting metalenses and meta-axicons for focusing and beaming of spontaneous emission

**DOI:** 10.1038/s41467-021-23433-0

**Published:** 2021-06-14

**Authors:** Yahya Mohtashami, Ryan A. DeCrescent, Larry K. Heki, Prasad P. Iyer, Nikita A. Butakov, Matthew S. Wong, Abdullah Alhassan, William J. Mitchell, Shuji Nakamura, Steven P. DenBaars, Jon. A. Schuller

**Affiliations:** 1 Department of Electrical and Computer Engineering, University of California Santa Barbara, CA, USA; 2 Department of Physics, University of California Santa Barbara, CA, USA; 3 Materials Department, University of California Santa Barbara, CA, USA; 4 Solid State Lighting and Energy Electronics Center, University of California Santa Barbara, CA, USA; 5 Nanofabrication Facility, University of California Santa Barbara, CA, USA

**Keywords:** Metamaterials, Nanophotonics and plasmonics

## Abstract

Phased-array metasurfaces have been extensively used for wavefront shaping of coherent incident light. Due to the incoherent nature of spontaneous emission, the ability to similarly tailor photoluminescence remains largely unexplored. Recently, unidirectional photoluminescence from InGaN/GaN quantum-well metasurfaces incorporating one-dimensional phase profiles has been shown. However, the possibility of generating arbitrary two-dimensional waveforms—such as focused beams—is not yet realized. Here, we demonstrate two-dimensional metasurface axicons and lenses that emit collimated and focused beams, respectively. First, we develop off-axis meta-axicon/metalens equations designed to redirect surface-guided waves that dominate the natural emission pattern of quantum wells. Next, we show that photoluminescence properties are well predicted by passive transmission results using suitably engineered incident light sources. Finally, we compare collimating and focusing performances across a variety of different light-emitting metasurface axicons and lenses. These generated two-dimensional phased-array photoluminescence waveforms facilitate future development of light sources with arbitrary functionalities.

## Abstract (web summary)

Controlling radiation emission using metamaterials is of interest for light sources and other applications. Here, the authors develop generalized phased-array metasurface equations and design metasurface axicons and lenses to collimate and focus the spontaneous emission, respectively.

## Introduction

Metasurface beam deflectors^1–3^, lenses^4–7^, axicons^7–9^, polarimeters^10,11^, vortex-beam generators^12,13^, and holograms^14,15^ highlight the capacity of metasurfaces for essentially arbitrary control of electromagnetic waveforms generated by spatially coherent sources, i.e., lasers. Light-emitting diodes (LEDs) are important technological light sources that emit light with low spatial coherence. As a result, many emerging LED technologies—such as optical neurostimulators^16^ and optical communications^17^—necessitate greater control over the spontaneous emission radiation pattern than what is currently demonstrated in the literature. Early studies of quantum dots coupled to plasmonic Yagi–Uda nanonatennas^18,19^ or plasmonic metasurfaces^20–22^ demonstrate the possibility to direct photoluminescence (PL) via phasing effects. Subsequent studies of luminescent metasurfaces have mostly comprised uniform arrays that modify and enhance spectra and directivity using resonant nanoantenna structures that are subwavelength in all dimensions^23–28^. However, these demonstrations lack the 2π phase range, amplitude control, and spatially extended phasing needed to achieve the robust wavefront control typical of passive metasurfaces. In a phased-array metasurface, the emission pattern is primarily controlled by the collective behavior of different meta-elements rather than the individual characteristic of the constituent meta-element in a uniform array. Recent studies of phase-gradient metasurfaces have demonstrated some degree of control over spontaneous emission^29–31^ and raised the yet-unrealized possibility of achieving generalized metasurface-mediated two-dimensional (2D) luminescence focusing.

Here, we show focusing and beaming of spontaneous emission using phased-array metasurfaces. We first develop generalized phased-array metasurface design concepts for redirecting traveling surface waves dominating the emission pattern of InGaN/GaN quantum wells. We then show how spontaneous emission properties can be predicted via transmission measurements with engineered incident light sources. We show that collimating meta-axicons increase the photon extraction and overall directivity. We demonstrate that metalenses focus the emitted PL at the designed focal lengths with beam widths that are inversely proportional to the numerical aperture. These results pave the way for new light sources where photons can be generated and redirected within the same compact space.

## Results

### Principles of the metasurface design

**Fig. 1:**
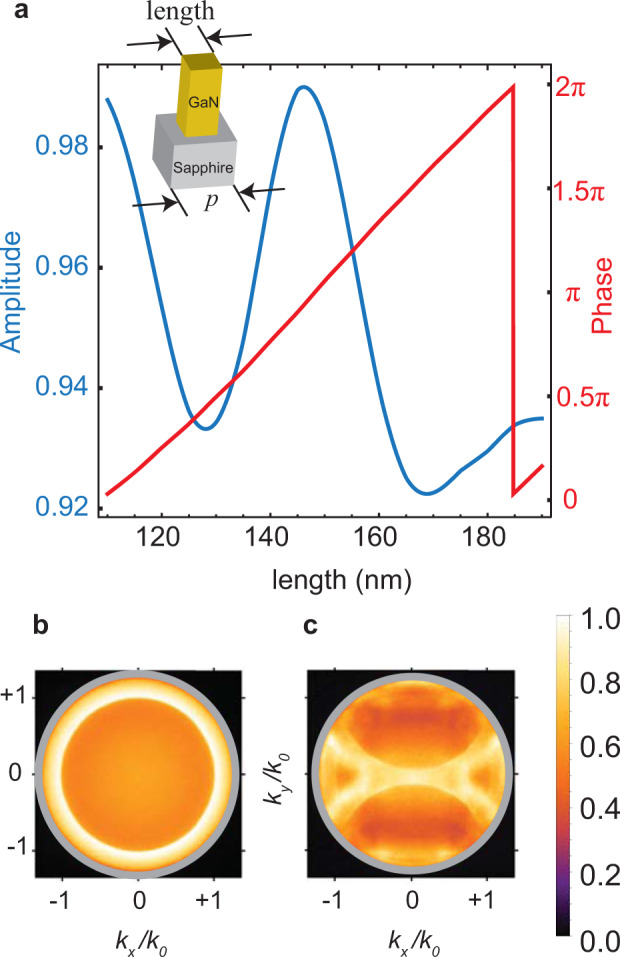
Design principles for light-emitting metasurfaces. **a** The transmitted amplitude (blue) and relative phase (red) of a plane wave impinging upon a GaN nanopillar from the top at a wavelength of 560 nm (Methods). The microperiod, *p*, of the structure is 250 nm. **b** Normalized intensity of measured unpolarized photoluminescence (PL) from the InGaN/GaN thin film as a function of normalized momenta ${k}_{x}/{k}_{0}$ and ${k}_{y}/{k}_{0}$. **c** Normalized intensity of measured unpolarized PL from an emitting 1D beam deflector, designed to impart a momentum of ${{k}_{y}=+0.8k}_{0}$ to the emission, as a function of normalized momenta ${k}_{x}/{k}_{0}$ and ${k}_{y}/{k}_{0}$. The gray ring corresponds to NA = 1.3.

Figure 1a shows the constituent element of our light-emitting metasurfaces. Metasurfaces comprise nonuniform arrays of 1-μm-tall, square cross-section, GaN nanopillars with embedded InGaN quantum wells located 100 nm below the surface (Methods). The phase of transmitted light at the quantum well peak emission wavelength (560 nm) is varied between 0 to $2\pi$ by changing the nanopillar lengths (Fig. 1a). This phase-length relationship is used to program desired spatial phase profiles. Consider, for instance, a metasurface that deflects an incident beam into a unidirectional diffraction lobe, defined equivalently by the output angle or in-plane momentum, $\left|{k}_{{\rm{||}},{\rm{out}}}\right|=\frac{2\pi n}{{\lambda }_{0}}{\rm{sin }}\left[{\theta }_{{\rm{out}}}\right]$, where *n* is the bulk refractive index of the output medium, ${\lambda }_{0}$ is the free-space wavelength, and ${\theta }_{{\rm{out}}}$ is the angle of exitance of the diffraction lobe. This is typically achieved with a 1D phase gradient^1,2^— ${\varphi }_{1{\rm{D}}}\left({\bf{r}}\right)=\left({{\bf{k}}}_{{\rm{||}},{\rm{out}}}-{{\bf{k}}}_{{\rm{||}},{\rm{in}}}\right)\cdot {\bf{r}}={\boldsymbol{\triangle }}{\bf{k}}\cdot {\bf{r}}$ — acting on a normal incidence input (${k}_{{\rm{||}},{\rm{in}}}=0$). Spontaneous emission, on the other hand, is naturally spread across the 2D momentum space and cannot rigorously be identified with a single input momentum. However, as seen in the back-focal-plane (BFP) image in Fig. 1b, the unpatterned quantum well emission is strongly concentrated just beyond the critical angle (at $\left|{k}_{{\rm{||}}}\right|=1.06{k}_{0}$, where ${k}_{0}$ is the wavenumber in free space). As a result, the photoluminescence (PL) from phase-gradient metasurfaces exhibits narrow 1D emission lobes (Fig. 1c) directed along the phase-gradient axis ($y$) according to

1 $${{\bf{k}}}_{{\rm{||}},{\rm{out}}}.\hat{{\bf{y}}}=\frac{\partial \varphi }{\partial y}+{k}_{{\rm{||}},{\rm{in}}}$$

where, we assume that the momentum of the incident wave (i.e., the generated PL) is ${k}_{{\rm{||}},{\rm{in}}}=-1.06{k}_{0}$ (a thorough discussion on choosing the proper momentum comes in subsequent sections). Below, we show how to generalize this 1D design heuristic to achieve 2D beam collimation and focusing in metasurface axicons and lenses, respectively.

### Meta-axicons

**Fig. 2:**
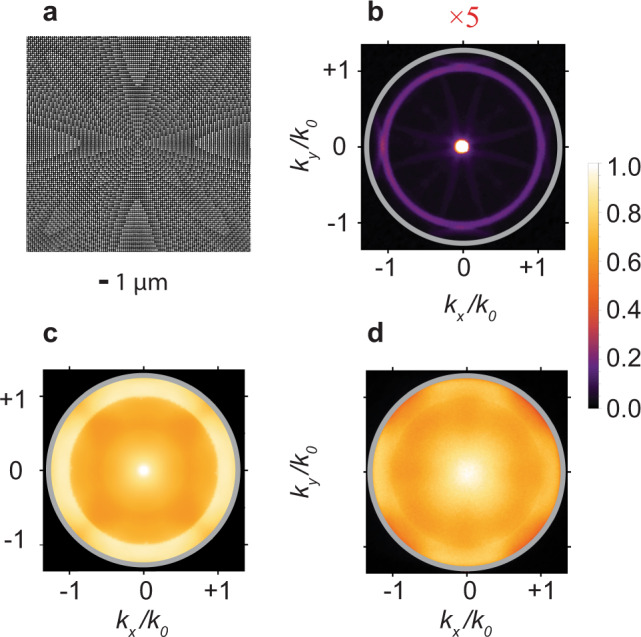
Luminescent meta-axicons for collimating ordinarily trapped spontaneous emission. **a** SEM image of the meta-axicon. **b** Back-focal-plane (BFP) image of the meta-axicon illuminated with a normally incident light at a wavelength of 560 nm. The bright spot at the center is the uncoupled incident light. The image is saturated by a factor of 5 to make the ring at $k=1.06{k}_{0}$, corresponding to the converted light, easily visible. **c** Theoretically derived, unpolarized emission of the light-emitting meta-axicon. The incident light used for this calculation is the calculated BFP of the InGaN/GaN thin film. **d** Measured unpolarized emission pattern of the light-emitting meta-axicon. The gray ring corresponds to NA = 1.3.

Axicons are one of the earliest studied examples of a 2D phased-array metasurface. Axicons can generate Bessel beams that exhibit properties such as non-diffraction and optical pulling forces^8^. Typical zero-order axicons (those that create zeroth-order Bessel beams) are defined by a linear phase gradient, along all radial directions: $\varphi \left({\bf{r}}\right)=\triangle k\times r$ (Fig. 2a), where $r$ is the radial distance along the surface from the meta-axicon center. As seen in Fig. 2b, they thus convert a collimated normal incident beam (${k}_{|{\rm{|}},{\rm{in}}}=0$) into a ring of illumination (${{\bf{k}}}_{{\rm{||}},{\rm{out}}}\left({\bf{r}}\right)=\triangle k\hat{{\bf{r}}}$). When $\triangle k=1.06{k}_{0}$, this ring of illumination coincides with the peaked local density of optical states (LDOS) for emission from unpatterned quantum wells (Fig. 1b). Applying principles of time-reversal symmetry, we might expect that same axicon to redirect this ordinarily trapped spontaneous emission toward normal exitance, enhancing the photon extraction and beam collimation. We construct a simple analytical model for this process by taking the calculated LDOS of the thin film, superimposing it with the diffraction mirrors defined by the square lattice (i.e., as if it were a uniform array, described in Supplementary Note 3), assuming equal amplitude for the diffracted modes and the zeroth order mode. We then take every point in this modified LDOS and surround it with a ring of light the amplitude of which is defined from the transmission measurement in Fig. 2b. From this measurement, we observe that 35% of the power is converted to the bright ring of light with a radius of $\triangle k=1.06{k}_{0}$ and 25% is converted to the four diffraction lobes adjacent to it (Fig. 2b). In this way, we calculate the BFP image produced by a meta-axicon at a single wavelength. To further improve the image accuracy, we account for the emission spectrum bandwidth (as shown in the Supplementary Fig. 3). We calculate the meta-axicon emission patterns at wavelengths between 535–605 nm in 10 nm increments. Lastly, we produce the final BFP image by taking a weighted sum of the single-wavelength images, with weights determined by the measured meta-axicon PL spectrum. A limitation of the current calculation is that we assume a constant conversion efficiency for every momentum and wavelength. Regardless, this simple model predicts the most prominent features of the BFP image, i.e., a clear bright spot at the center of the BFP image for the meta-axicon emission, shown in Fig. 2c, which is in good agreement with the experimental BFP image shown in Fig. 2d. Examples of other meta-axicons that impart different momenta to the emission (shown in Supplementary Fig. 4) indicate that the theoretical model reproduces the most prominent features of the experimental BFP image. The radial phase gradient successfully redirects a significant fraction of ordinarily trapped photons ($\left|{k}_{{\rm{||}}}\right|\, > \, {k}_{0}$) to near-normal exitance, increasing the photon extraction and beam collimation. In comparison to the corresponding thin films, the overall directivity (Supplementary Note 6) is enhanced by 38% (a directivity of 2.00 for the meta-axicon as opposed to a value of 1.45 for the thin film) and the fraction of photons emitted beyond the critical angle is reduced by 25%. We note that the relatively low conversion efficiency of 35% in Fig. 2b is due to the large momentum that the meta-axicon imparts to the incident light ($\triangle k=1.06{k}_{0}$). The larger the imparted momentum, the lower the conversion efficiency. For example, for a $\triangle k=0.7{k}_{0}$ meta-axicon, a maximum efficiency of 55% is reported^8^. This is a well known phenomenon in passive phased-array metasurfaces^8^ which can be remedied by using more complex unit cells^32,33^.

Earlier works have demonstrated beam collimation via uniform nanocylinder arrays^34–36^. In these works, the design procedure is relatively ad hoc: the nanocylinder diameters are varied within a range to find the diameter that best generates a collimated beam. In this approach, the emission pattern is mainly determined by the individual characteristic of a single nanocylinder. In a phased-array metasurface, i.e., a meta-axicon, however, the emission pattern is primarily controlled by the collective behavior of nanocylinders of different diameters that cover a $2\pi$ phase range^1,37^. An obvious indication is that meta-axicons with different phase gradients produce drastically different BFP images (Fig. 2 and Supplementary Fig. 4), showing normal exitance collimation only when the correct phase gradient is applied (Fig. 2). This unambiguously indicates that the observed collimation effect in Fig. 2 is a phased-array collimation and not a collimation introduced by an individual nanopillar. Interestingly, the range of diameters used to achieve the $2\pi$ phase coverage is not unique, and better collimation may be possible by combining the uniform-array effects described above with the phased-array effects shown here.

### Metalenses

**Fig. 3:**
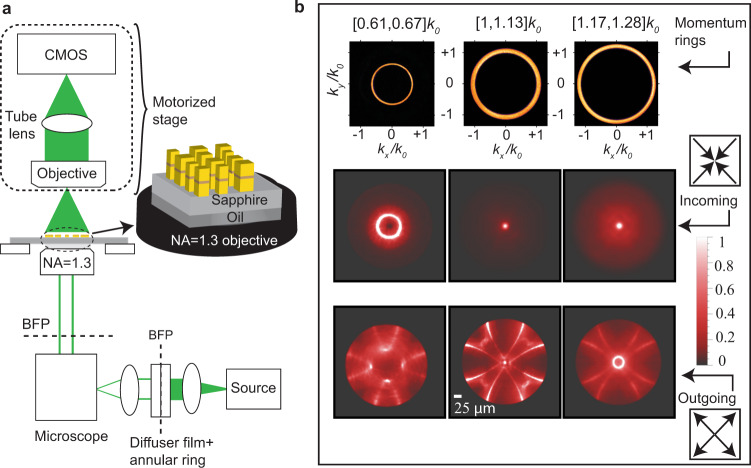
Understanding luminescent metalens functionality through structured illumination. **a** The measurement setup used to characterize the focusing performance of the metalenses in transmission, once the excitation is a monochromatic, oblique plane wave at a wavelength of 560 nm. **b** Metalenses are illuminated with three different momentum-space rings: $0.61{k}_{0}$ < $\left|{k}_{{\rm{|}}{{\rm{|}}}i}\right|\, <\, 0.67{k}_{0}$; ${k}_{0}$ < $\left|{k}_{{\rm{|}}{{\rm{|}}}i}\right|\, <\, 1.13{k}_{0}$; and $1.17{k}_{0}$ < $\left|{k}_{{\rm{|}}{{\rm{|}}}i}\right|\, <\, 1.28{k}_{0}$. The real-space focal profiles of metalenses designed to have a focal length of $f=150{\,{\rm{\mu }}}$m are shown under illumination with these three momentum rings, for both the “incoming” and “outgoing” metalens designs. The “incoming” and “outgoing” sketches and their accompanying arrows show the direction of the traveling waves on the surface of the metalens with respect to the center of the metalens.

The demonstration of collimated PL from light-emitting meta-axicons confirms that our derived 1D design heuristic, which assumes an incident momentum of $|{{k}_{{\rm{||}}}}_{{\rm{i}}}|=1.06{k}_{0}$, instead of the commonly used $|{{k}_{{\rm{||}}}}_{{\rm{i}}}|=0$, can be generalized to radially symmetric 2D phase patterns. Applying these principles to metasurface lenses, we develop an “off-axis” phased-array lens equation (Supplementary Note 4) for focusing PL:

2 $${\varphi }_{{\rm{lens}}}\left(r\right)=-{k}_{0}n\left(\sqrt{{f}^{2}+{r}^{2}}-f\right)\pm 1.06{k}_{0}r$$

where, $f$ is the design focal length. The critical difference between this expression and the conventional metalens phase profile lies in the second term in Eq. (2). This term accounts for the phase evolution of oblique waves propagating radially along the metasurface toward (+) or away (−) from the metalens center. Either choice of sign will lead to focusing of the targeted ray, but, as we will show, with different focusing quality. This can be inferred directly from transmission measurements using a momentum-engineered incident light source. Using BFP illumination techniques (Methods), we illuminate our metalenses with monochromatic light ($\lambda =560$ nm) comprising an annulus in the momentum space (Fig. 3). This illumination technique is intended to imitate the natural emission pattern of the quantum wells (e.g., Fig. 1b) and help explain the PL images produced by the metalenses. We first consider an annulus defined by ${k}_{0}\, <\, |{{k}_{{\rm{||}}}}_{{\rm{i}}}|\, <\, 1.13{k}_{0}$ (Fig. 3). Metalenses employing either choice of sign exhibit a focused beam at the desired focal length (Fig. 3b). However, due to steeper phase gradients, the “outgoing (−)” metalens exhibits poorer focusing efficiency (Supplementary Note 7) and significantly brighter diffractive signatures: the central focus appears near the intersection of four bright circular arcs. We next consider the impact of rays even further beyond the critical angle. When illuminated by rays with ${1.17k}_{0}\, <\, |{{k}_{{\rm{||}}}}_{{\rm{i}}}|\, <\, 1.28{k}_{0}$, the “incoming (+)” metalens focal spot expands, while the “outgoing (−)” metalens focal spot evolves into a ring. Interestingly, when we illuminate the metalenses by rays with ${0.61k}_{0}\, <\, |{{k}_{{\rm{||}}}}_{{\rm{i}}}|\, <\, 0.{67k}_{0}$, the focal spot for the “incoming” metalens turns into a ring and the “outgoing” metalens shows no clear focusing (Fig. 3b).

## Discussion

**Fig. 4:**
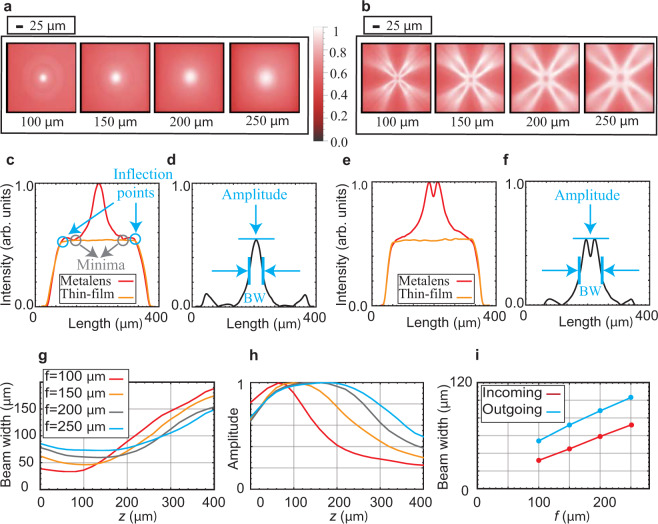
Focused photoluminescence (PL) from emitting metalenses. **a** Focal profiles of “incoming” metalenses. **b** Focal profiles of “outgoing” metalenses. **c** “Incoming” metalens and thin-film emission intensities along a horizontal central cut in their respective emission images. **d** The “incoming” metalens-mediated emission intensity derived by subtracting the scaled thin film emission from the metalens emission. **e** “Outgoing” metalens and thin-film emission intensities along a horizontal central cut in their respective emission images. **f** The “outgoing” metalens-mediated emission intensity derived by subtracting the scaled thin film emission from the metalens emission. **g** Evolution of beam widths (“BW” in panels **d**, **f**) as a function of *z* (depth into the focal region) for “incoming” metalenses. **h** Amplitude evolution as a function of *z* for “incoming” metalenses, normalized to the corresponding maximum value for each metalens. **i** Beam width values at the focal plane as a function of the designed focal length for both the “incoming” and “outgoing” metalenses.

At the designed focal lengths, both “incoming (+)” and “outgoing (−)” metalenses form PL images (Fig. 4). The evolution of PL images with depth for the “incoming” and “outgoing” metalenses are shown in Supplementary Figs. 7 and  8, respectively. For both metalens designs, PL images closely resemble those produced via passive illumination with momentum-structured beams (e.g., Fig. 3). The “incoming (+)” metalens exhibits superior focusing performance and is the focus of the subsequent analysis. The focused component lies on top of a relatively flat background, which is subtracted through comparison to unstructured thin films (Fig. 4 and Methods). Figure 4g, h show the evolution of the beam width (full-width half-max) and normalized amplitude as a function of focal depth for four metalenses. The point of minimal beam width is close to the target focal length for all metalenses. Beam widths as small as ~30 μm were achieved in the shortest focal length metalens. The beam width increases as focal length increases, consistent with conventional diffraction-limited focusing (Fig. 4i). Further analysis of the metalenses is given in Supplementary Note 8. Interestingly, the beam focus is asymmetric; that is the beam width remains relatively constant until the focal point, after which it starts to diverge significantly. This behavior, however, follows from the passive transmission results, where higher momenta rays converge before the designed metalens focus.

The results presented here demonstrate collimation and focusing of PL using generalized phased-array metasurface concepts developed for incoherent emission. We designed meta-axicons and metalenses around highly-oblique surface-like waves that dominate the LDOS of unstructured films. We demonstrate meta-axicons that redirect emitted PL toward normal exitance to increase photon extraction and show how these results can be predicted from transmission experiments. Moreover, we design and fabricate eight different metalenses and characterize their behavior under structured illumination. We then show that these metalenses focus PL at the target focal length with beam widths that are inversely proportional to the numerical aperture. These results pave the way for light-emitting metasurfaces with programmable functionalities where photons are generated and redirected within the same compact space. LED displays, Li-Fi, solid-state lighting, and optogenetics are among the potential beneficiaries of the design concepts developed here. In fact, the developed concepts are wavelength-agnostic and may be used to further control the incoherent thermal emission from IR sources as well, which is an area of active research. Future iterations may employ more sophisticated elements or multi-layer geometries^38^ to increase efficiency or unlock more complex beam-forming possibilities.

## Methods

### Growth

Samples were grown hetero-epitaxially on (0001) c-plane double-side-polished sapphire substrates by atmospheric pressure metal-organic chemical vapor deposition (MOCVD). The whole structure consisted of an AlGaN nucleation layer, an $\sim$0.85-µm unintentionally doped (UID) GaN layer, three-period multiple quantum wells (MQWs) with a 3 nm InGaN QW, a 2 nm Al0.3 Ga0.7 N cap, and a 10 nm GaN barrier. The InGaN layers were grown at 760 °C, and the GaN and AlGaN layers were grown at 835 °C. Finally, a 100 nm UID GaN protective buffer is grown on top to serve as a protection layer for the emitting QW from fabrication damages. The distance between the quantum well layer and the GaN-air interface is chosen such that the quantum wells are located at the anti-node of standing waves formed in the thin film, resulting in efficient light emission. In addition, the distance between the quantum well layer and the GaN-sapphire interface is chosen to be several wavelengths long. This makes the electromagnetic waves emitted by the quantum wells toward the substrate experience a phase coverage of $2\pi$ across the range of nanopillars lengths shown in Fig. 1a. If, however, one wants to modify the PL emission toward air instead of sapphire, then the quantum wells should be several wavelengths away from the GaN-air interface to provide the $2\pi$ phase coverage needed for efficient operation of the phased-array metasurfaces.

### Fabrication

Samples were ultrasonically cleaned with acetone and isopropanol for organic contaminants removal, before processing. Subsequently, 420 nm of SiO_2_ was deposited on the samples, using an Advanced Vacuum PECVD system, to serve as the hard mask for the GaN etch. Sputtering was used to deposit a 32-nm-thick ruthenium layer on top of the SiO_2_ layer to serve as the hard mask for the SiO_2_ etch. A 20-second O_2_ descum was performed in an O_2_ barrel asher at 300 mT and 100 W to increase the ruthenium surface stiction prior to resist spinning. Subsequently, a 50-nm-thick layer of hydrogen silsesquioxane (HSQ) was spun on top and baked at 100 °C for 45 s to be used as a negative resist for electron beam lithography. Following the exposure, the sample was put in a 25% tetramethylammonium hydroxide (TMAH) solution for 60 s for developing and then rinsed with DI water. The ruthenium hard mask was removed in an inductively coupled plasma (ICP) etcher (18.8 mT, O_2_ at 49.5 sccm, Cl_2_ at 5.5 sccm, high-frequency power 50 W, ICP power 500 W). The SiO_2_ hard mask was removed in a Fluorine ICP etcher (5.0 mT, CF_4_ at 50.0 sccm, CHF_3_ at 12.5 sccm, high-frequency power 40 W, ICP power 950 W). Before performing the GaN etch, we removed the leftover ruthenium using the same ruthenium etch mentioned earlier. GaN was etched in an ICP etcher (6.8 mT, Cl_2_ at 38 sccm, N_2_ at 14 sccm, high-frequency power 200 W, ICP power 500 W). This low-pressure etch recipe almost uniformly etches the nanopillars of different sizes throughout the sample and is extremely vertical. The remaining SiO_2_ hard mask was removed by dipping the sample in buffered hydrofluoric acid (HF) for 120 s.

### FDTD simulations

We performed the simulations using a commercial package from Lumerical Inc. (FDTD Solutions). A unit cell consisting of 1-µm-tall GaN nanopillars (refractive index of 2.28) on top of a sapphire substrate (refractive index of 1.77) was simulated. The nanopillars were 1 μm tall in the *z* direction. Periodic boundary conditions were considered along the *x* and *y* directions, and perfectly matched layers (PML) were used along the *z* direction. Nanopillars were placed at the center of the unit cell. The period of this square unit cell was 250 nm. Simulations were performed with the assumption of a plane wave that is propagating along the *z* direction. A minimum mesh size of 2, 2, and 4 nm was considered along the *x*, *y*, and *z* directions, respectively. The length of the nanopillar was varied and the transmission phase and amplitude inside the substrate were monitored. This phase and amplitude as a function of nanopillar size, shown in Fig. 1a, was used for designing the metasurfaces.

### Passive transmission measurements

All passive transmission measurements of metalenses with BFP illumination, shown in Fig. 3, were performed using a monochromatic light source at a wavelength of 560 nm. These transmission measurements are intended to imitate the natural emission pattern of our emitting structure (e.g., Fig. 1b) before structuring, which consists primarily of a ring of intensity around 1.06*k*_0_. We illuminated metasurfaces with waves with transverse momentum values lower than, equal to, and higher than that of the design momentum ($1.06{k}_{0}$). These measurements were performed using a home-built momentum-resolved system.

Metalenses were designed assuming an incident wave with a momentum of ${\rm{|}}{{k}_{{\rm{||}}}}_{{\rm{i}}}{\rm{|}}=1.06{k}_{0}$, corresponding to a ring of light in the momentum space. To accomplish this, we place a transmitting annulus in the objective’s conjugate BFP, i.e., allowing light with certain momenta, ${\rm{|}}{{k}_{{\rm{||}}}}_{i}{\rm{|}}$, to pass (e.g., Fig. 3b). The annulus was uniformly illuminated with a monochromatic source (wavelength of 560 nm). Three separate experiments were performed, one with a ring of light corresponding to $0.61{k}_{0}\, <\, |{{k}_{{\rm{||}}}}_{{\rm{i}}}{\rm{|}}\, <\, 0.67{k}_{0}$, one corresponding to ${k}_{0}\, <\, |{{k}_{{\rm{||}}}}_{{\rm{i}}}{\rm{|}}\, <\, 1.13{k}_{0}$, and the other corresponding to ${1.17k}_{0}\, <\, |{{k}_{{\rm{||}}}}_{{\rm{i}}}{\rm{|}}\, <\, 1.28{k}_{0}$, as shown in Fig. 3. The average momenta of these rings are $0.64{k}_{0}$ (lower than the design momentum), $1.06{k}_{0}$ (the design momentum), and $1.22{k}_{0}$ (higher than the design momentum), respectively. The collection setup consists of a 10X objective lens (Nikon 10× 0.3 NA Plan Fluor) in conjunction with a singlet lens (focal length *f* = 200 mm), resulting to an equivalent NA = 0.12, as shown in Fig. 3. The collected light is then imaged with a CMOS camera (DCC1545M CMOS Camera, ThorLabs). Scanning was achieved by a motorized stage (Newport). The intensity profiles at different heights were recorded by the camera to obtain the transmission profile created by each metalens.

### Calculation of beam widths for the emitting metalenses

We follow the procedure shown in Fig. 4c–f to calculate the beam widths. In order to calculate the beam width, we consider horizontal linecuts of the real-space images and find the inflection points of the intensity (second derivative dropping to zero), for both the metalens and the thin film (Fig. 4). We then normalize the magnitudes of the intensity cuts such that the intensities are equal at the inflection point. We consider the thin film intensity as a “background” or “reference” to represent the behavior of spontaneously emitted light in the absence of structuring, and subtract the thin film intensity from the metalens intensity. The resulting information allows us to quantify both the degree to which the metasurface redistributes this light, and the metalens beam widths (e.g., Fig. 4d,f). Beam widths for the “incoming” and “outgoing” metalenses were defined differently, as described in Fig. 4d, f, respectively.

## Supplementary information

Supplementary Information

## Data Availability

The data that support the plots within this paper and other findings of this study are available from the corresponding author upon reasonable request.

## Code availability

The codes that support the plots within this paper are available from the corresponding author upon reasonable request.

## References

[1] Yu N, Capasso F (2014). Flat optics with designer metasurfaces. Nat. Mater..

[2] Yu N (2011). Light propagation with phase discontinuities: generalized laws of reflection and refraction. Science.

[3] Wang S (2017). Broadband achromatic optical metasurface devices. Nat. Commun..

[4] Khorasaninejad M (2016). Metalenses at visible wavelengths: diffraction-limited focusing and subwavelength resolution imaging. Science.

[5] Chen WT (2018). A broadband achromatic metalens for focusing and imaging in the visible. Nat. Nanotech..

[6] Zhang S (2016). High efficiency near diffraction-limited mid-infrared flat lenses based on metasurface reflectarrays. Opt. Express.

[7] Aieta F (2012). Aberration-free ultrathin flat lenses and axicons at telecom wavelengths based on plasmonic metasurfaces. Nano Lett..

[8] Chen W (2017). Generation of wavelength-independent subwavelength Bessel beams using metasurfaces. Light Sci. Appl..

[9] Lin D, Fan P, Hasman E, Brongersma ML (2014). Dielectric gradient metasurface optical elements. Science.

[10] Chen WT (2016). Integrated plasmonic metasurfaces for spectropolarimetry. Nanotechnology.

[11] Pors A, Nielsen MG, Bozhevolnyi SI (2015). Plasmonic metagratings for simultaneous determination of Stokes parameters. Optica.

[12] Yue F (2016). Vector vortex beam generation with a single plasmonic metasurface. ACS Photonics.

[13] Zhang Y, Gao J, Yang X (2019). Spatial variation of vector vortex beams with plasmonic metasurfaces. Sci. Rep..

[14] Zheng G (2015). Metasurface holograms reaching 80% efficiency. Nat. Nanotech..

[15] Li X (2016). Multicolor 3D meta-holography by broadband plasmonic modulation. Sci. Adv..

[16] Bi X (2016). A flexible, micro-lens-coupled LED stimulator for optical neuromodulation. IEEE Trans. Biomed. Circuits Syst..

[17] Rein M (2018). Diode fibres for fabric-based optical communications. Nature.

[18] Curto AG (2010). Unidirectional emission of a quantum dot coupled to a nanoantenna. Science.

[19] Kosako T, Kadoya Y, Hofmann HF (2010). Directional control of light by a nano-optical Yagi-Uda antenna. Nat. Photonics.

[20] Langguth L, Schokker AH, Guo K, Koenderink AF (2015). Plasmonic phase-gradient metasurface for spontaneous emission control. Phys. Rev. B.

[21] Hancu IM, Curto AG, Castro-López M, Kuttge M, van Hulst NF (2013). Multipolar interference for directed light emission. Nano Lett..

[22] Park Y (2017). Metasurface electrode light emitting diodes with planar light control. Sci. Rep..

[23] Liu S (2018). Light-emitting metasurfaces: simultaneous control of spontaneous emission and far-field radiation. Nano Lett..

[24] Staude I (2015). Shaping photoluminescence spectra with magnetoelectric resonances in all dielectric nanoparticles. ACS Photonics.

[25] Yuan S (2017). Strong photoluminescence enhancement in all-dielectric Fano metasurface with high quality factor. ACS Nano.

[26] Bucher T (2019). Tailoring photoluminescence from MoS2 monolayers by Mie-resonant metasurfaces. ACS Photonics.

[27] Capretti A, Lesage A, Gregorkiewicz T (2017). Integrating quantum dots and dielectric Mie resonators: a hierarchical metamaterial inheriting the best of both. ACS Photonics.

[28] Vaskin A, Kolkowski R, Koenderink AF, Staude I (2019). Light-emitting metasurfaces. Nanophotonics.

[29] Iyer PP (2020). Unidirectional luminescence from InGaN/GaN quantum-well metasurfaces. Nat. Photonics.

[30] Khaidarov E (2019). Control of LED emission with functional dielectric metasurfaces. Laser Photonics Rev..

[31] Rong K (2020). Photonic Rashba effect from quantum emitters mediated by a Berry-phase defective photonic crystal. Nat. Nanotechnol..

[32] Sell D, Yang J, Doshay S, Yang R, Fan JA (2017). Large-angle, multifunctional metagratings based on freeform multimode geometries. Nano Lett..

[33] Arbabi A (2020). Increasing efficiency of high numerical aperture metasurfaces using the grating averaging technique. Sci. Rep..

[34] Van Dam D (2015). Directional and polarized emission from nanowire arrays. Nano Lett..

[35] Paniagua-Domínguez R, Grzela G, Rivas JG, Sánchez-Gil JA (2013). Enhanced and directional emission of semiconductor nanowires tailored through leaky/guided modes. Nanoscale.

[36] Van Dam D (2016). Strong diameter-dependence of nanowire emission coupled to waveguide modes. Appl. Phys. Lett..

[37] Arbabi A, Horie Y, Bagheri M, Faraon A (2015). Dielectric metasurfaces for complete control of phase and polarization with subwavelength spatial resolution and high transmission. Nat. Nanotech.

[38] Arbabi A (2016). Miniature optical planar camera based on a wide-angle metasurface doublet corrected for monochromatic aberrations. Nat. Commun..

