# Supplementary material for: Light-emitting metalenses and meta-axicons for focusing and beaming of spontaneous emission
Source: Nat Commun. 2021 Jun 14;12:3591. doi: 10.1038/s41467-021-23433-0 (PMC8203637; doi:10.1038/s41467-021-23433-0)
Supplement: Supplementary file 1 — Supplementary Information [file 41467_2021_23433_MOESM1_ESM.pdf]

Supplementary Information

Light-emitting metalenses and meta-axicons for focusing and beaming of spontaneous emission

Yahya Mohtashami[1], Ryan A. DeCrescent[2], Larry K. Heki[3], Prasad P. Iyer[1], Nikita A. Butakov[1], Matthew S. Wong[3,4], Abdullah Alhassan[3,4], William J. Mitchell[5], Shuji Nakamura[1,3,4], Steven P. DenBaars[1,3,4], and Jon. A. Schuller[1,*]
1. Department of Electrical and Computer Engineering, University of California Santa Barbara
2. Department of Physics, University of California Santa Barbara
3. Materials Department, University of California Santa Barbara
4. Solid State Lighting and Energy Electronics Center, University of California Santa Barbara,
5. Nanofabrication Facility, University of California Santa Barbara

Supplementary Note 1. Measuring far-field emission pattern with back-focal-plane-imaging

We measure the emission patterns of the light-emitting metasurfaces by employing Fourier microscopy[1,2] by re-imaging the back-focal-plane of an oil-immersion objective with a NA=1.3 (Nikon 100x 1.3 NA Plan Fluor) to an imaging spectrometer (Princeton Instruments Iso Plane SCT320 with Princeton Instruments PIXIS 1024BRX), shown in Supplementary Fig. 1. We pump the samples by a 405-nm light-emitting diode (LED) (M405L3, ThorLabs). The LED output is then passed through diffuser films to uniformly illuminate the emitting surface. This diffused incident light was filtered via a 405-nm short-pass filter (Semrock) to make sure that any possible long-wavelength components of the LED are effectively suppressed. The output of the filter is then reflected from a dichroic mirror (Semrock) and redirected toward the sample. The emitted photoluminescence (PL) from the sample passes through the same dichroic mirror, as well as a 417-nm long-pass filter (Semrock). This emitted PL exits the output port of the microscope and

passes through a Bertrand lens. This gives us the 2D momentum-space distribution (k_x, k_y) of the emitted PL from the sample.

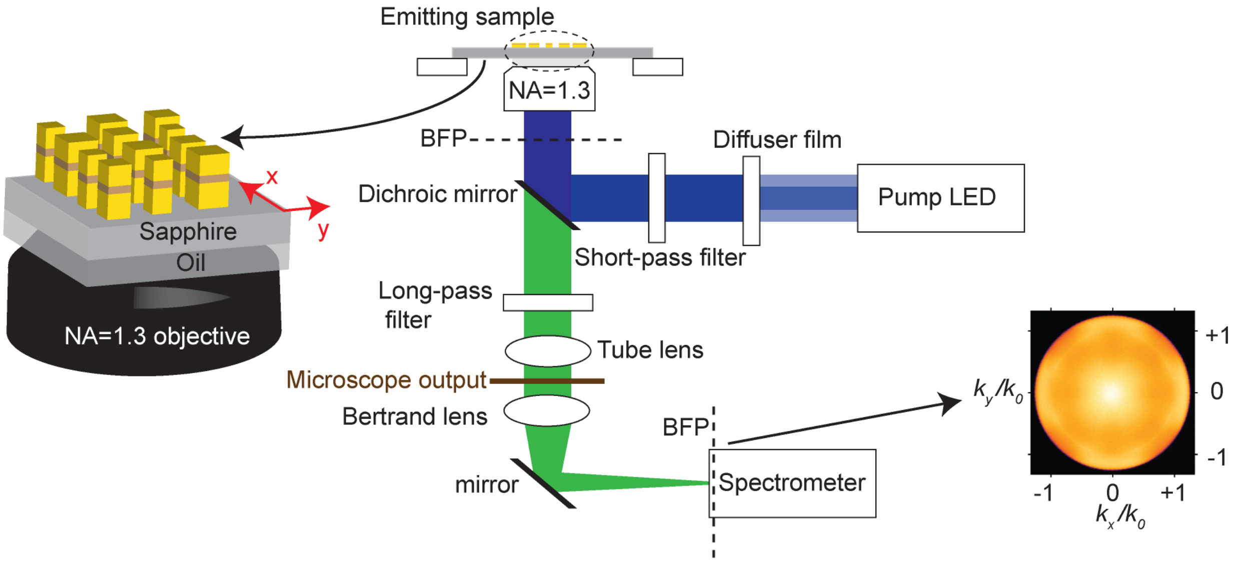

Supplementary Fig. 1: Momentum-resolved photoluminescence (PL) measurement setup performed with a high-NA oil-immersion objective. This setup gives us the emission pattern of the light-emitting metasurface in the momentum space. The output 405-nm light from the diffuser is a diffused beam but is drawn as a collimated beam for simplicity. The microscope output port is shown in dark brown.

Supplementary Note 2. Spatially resolved imaging of the emitted PL from the metalenses

To perform spatially resolved imaging to characterize the performance of the metalenses, we use the same excitation and filter set as the ones shown in Supplementary Fig. 1; however, a different collection setup from the one shown in Supplementary Fig. 1 is employed. We exchange the oil-immersion objective lens with a 20X objective lens (Nikon 20x 0.5 NA Plan Fluor) with a NA=0.5, as shown in Supplementary Fig. 2. The light emitted by the metalens is imaged by this 20X objective to the output of the microscope, and re-imaged using a 1:1 achromatic doublet pair (ThorLabs) to a CMOS camera (DCC1545M CMOS Camera, ThorLabs). The intensity profile at

each height (z) was recorded by scanning the objective, in increments of 2 µm, to focus at different planes through the 400-µm-thick sapphire substrate.

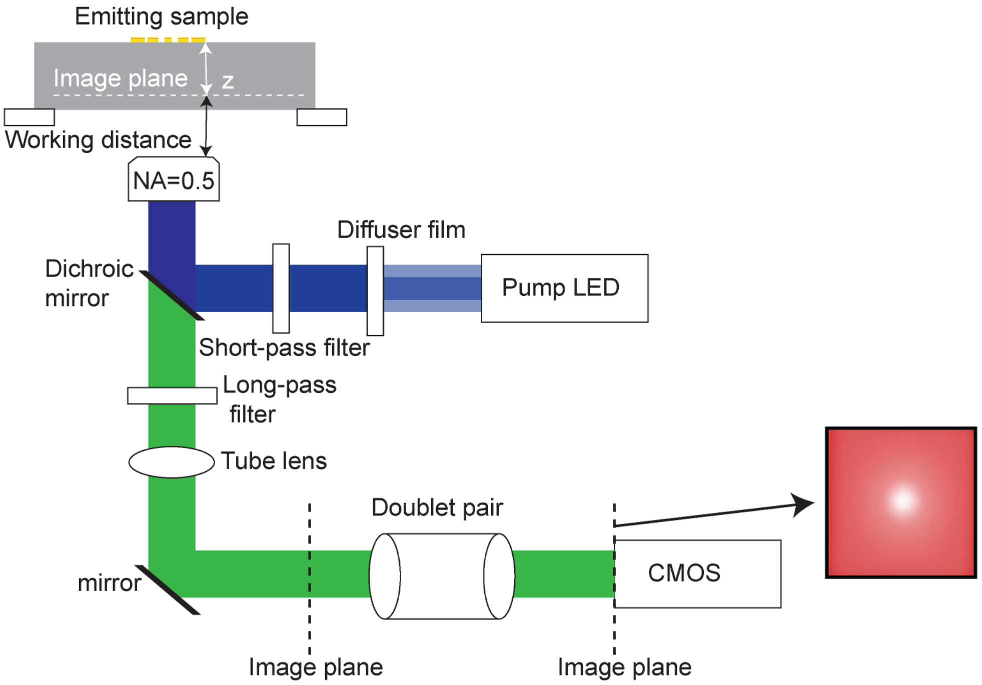

Supplementary Fig. 2: The experimental setup used for spatially resolved imaging of the emitted light by metalenses. An objective with a NA of 0.5 is used in these experiments. The output 405-nm light from the diffuser is a diffused beam, but it is drawn as a collimated beam for simplicity. The output of the doublet pair is drawn as a collimated beam for simplicity.

Supplementary Note 3. Effect of nanopatterning on the local density of optical states

Once a photonic crystal with a microperiod of p is etched onto the thin film, a lattice momentum of $G_0 = 2\pi/p$ couples the emission modes with a momentum of $\mathbf{k}$ to photonic crystal modes with momenta of $\mathbf{k} + mG_0\hat{\mathbf{x}} + nG_0\hat{\mathbf{y}}$ for any integer m and n. Thus, by nanopatterning, we are in effect re-imaging the BFP image of the thin film to a grid of BFP images centered at (mG_0, nG_0). The spacing between these images is G_0, which for a wavelength of 560 nm will be $2.18k_0$. The momentum at which the thin film emission peaks is $1.06k_0$, meaning that the $G_0/2$ value in fact coincides with the momentum of the peak emission intensity. Therefore, the neighboring BFP

images nicely overlap, further intensifying the emission at $1.06k_0$. If the microperiod of the structure is increased or decreased, the overlap will become less pronounced. Thus, if one has a thin film that has a strong emission at a momentum that is significantly different from $1.06k_0$, the microperiod should be adjusted such that the overlap between neighboring BFP images is maximized.

Supplementary Fig. 3 shows the PL intensity of the thin film and the collimating meta-axicon as a function of wavelength. Upon etching the structure, the wavelength that corresponds to the peak PL intensity blue-shifts from 580 nm to 563 nm.

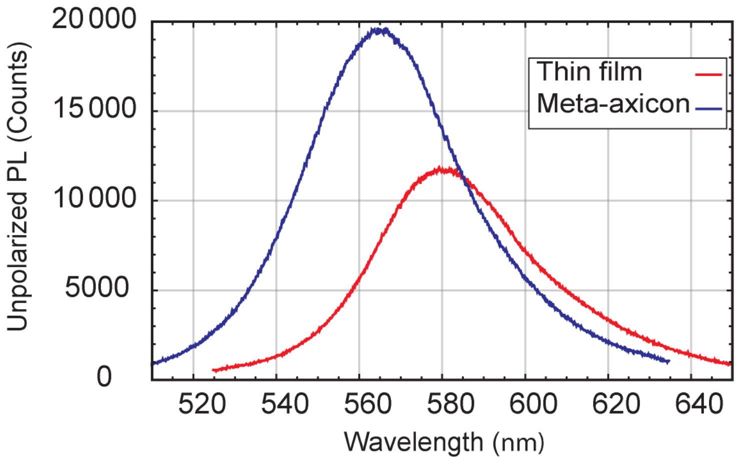

Supplementary Fig. 3: Photoluminescence spectra of the thin film and the collimating meta-axicon as a function of wavelength. The wavelength corresponding to the peak emission intensity blue-shifts upon etching the structure.

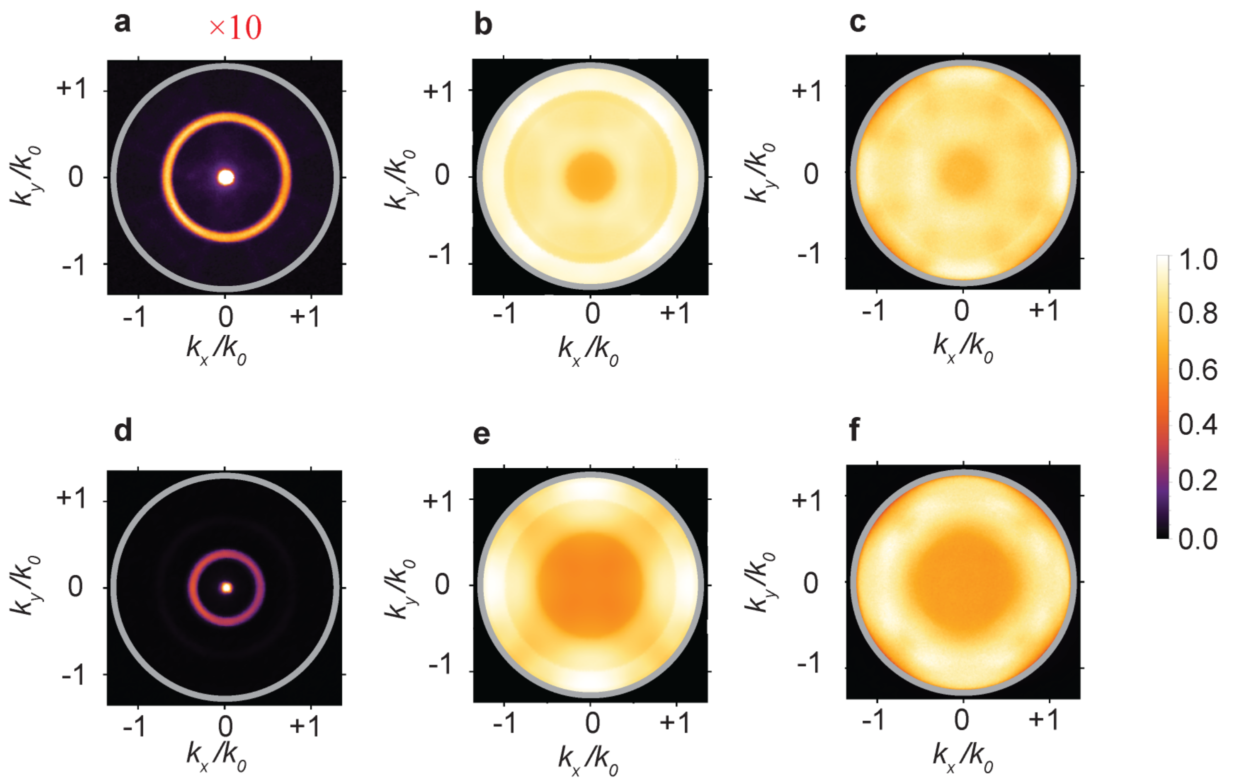

Supplementary Fig. 4: Transmission (left), calculated photoluminescence (middle), and experimental photoluminescence (right) back-focal-plane (BFP) images for meta-axicons with different phase gradients. **(a)** BFP image of a meta-axicon illuminated with a normally incident light at a wavelength of 560 nm. The bright spot at the center is the uncoupled incident light. The meta-axicon is designed with a phase gradient designed to produce a ring at $|k_{||}| = 0.7k_0$ for a normally incident light. The image is saturated by a factor of 10 to make this ring easily visible. **(b)** Theoretically calculated, unpolarized emission pattern of the light-emitting meta-axicon in panel a. **(c)** Measured unpolarized emission pattern of the light-emitting meta-axicon in panel a. **(d)** BFP image of a meta-axicon illuminated with a normally incident light at a wavelength of 560 nm. The bright spot at the center is the uncoupled incident light. The meta-axicon is designed with a phase gradient designed to produce a ring at $|k_{||}| = 0.4k_0$ for a normally incident light. **(e)** Theoretically calculated, unpolarized emission pattern of the light-emitting meta-axicon in panel d. **(f)** Measured unpolarized emission pattern of the light-emitting meta-axicon in panel d. The gray rings correspond to NA=1.3.

Supplementary Note 4. Generalized phase equations for meta-axicons and metalenses

Supplementary Fig. 5 illustrates the generalized law of refraction[3], where the incident light is anomalously refracted. The generalized law of refraction for Supplementary Fig. 5(a) can be written as

$$\frac{d\varphi(x)}{dx} = k_0 n_\mathrm{t} \sin(\theta_\mathrm{t}) - k_0 n_\mathrm{i} \sin(\theta_\mathrm{i}) = k_{||_\mathrm{t}} - k_{||_\mathrm{i}}, \tag{1}$$

where, $k_{||_\mathrm{t}}$ and $k_{||_\mathrm{i}}$ correspond to the transverse momenta in the media characterized, respectively, by indices of refraction n_t and n_i. In this equation, both angles and hence both transverse momenta are positive, because they are pointing in the same direction, i.e., $\hat{\mathbf{x}}$. This is not the case for Supplementary Fig. 5(b), however, since the anomalously refracted wave is pointing in the $-\hat{\mathbf{x}}$. Therefore, θ_t will adopt a negative value, while θ_i still remains positive. This results in a negative value for $k_{||_\mathrm{t}}$ and Supplementary Equation (1) will be modified for Supplementary Fig. 5(b) as

$$\frac{d\varphi(x)}{dx} = -k_0 n_\mathrm{t} \sin(|\theta_\mathrm{t}|) - k_0 n_\mathrm{i} \sin(\theta_\mathrm{i}) = -|k_{||_\mathrm{t}}| - k_{||_\mathrm{i}}, \tag{2}$$

where, we used the absolute value for both the angle and transverse momentum in the medium characterized by n_t to prevent confusion. Let us expand this even further and assume that we have an incident wave with the transverse momentum in the $-\hat{\mathbf{x}}$ direction. θ_i and $k_{||_\mathrm{i}}$ become negative values in Supplementary Equation (2) and we thus re-write the equation as $\frac{d\varphi(x)}{dx} = -k_0 n_\mathrm{t} \sin(|\theta_t|) + k_0 n_\mathrm{i} \sin(|\theta_\mathrm{i}|) = -|k_{||_\mathrm{t}}| + |k_{||_\mathrm{i}}|$. We then change the variable x to r (the radial coordinate) and assume that the incident wave is a surface wave with a momentum $|k_{||_i}|$=$1.06k_0$ traveling in the $-\hat{\mathbf{r}}$ direction ("incoming wave"), i.e., $\mathbf{k}_{||_\mathrm{i}} = -1.06k_0\hat{\mathbf{r}}$, that is to be deflected toward the center of the metasurface at an angle of $-|\theta_\mathrm{t}|$. We, thus, get

$$\frac{d\varphi(r)}{dr} = -k_0 n_\mathrm{t} \sin(|\theta_\mathrm{t}|) + 1.06k_0, \tag{S3}$$

which for $\theta_\mathrm{t} = 0$ will give the phase profile for a collimating meta-axicon in the incoming-wave scenario.

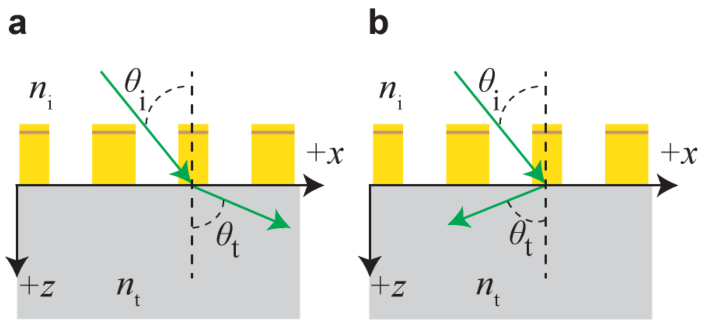

Supplementary Fig. 5: Generalized law of refraction for an obliquely incident light at an angle of θ_{i}. **(a)** Positive refraction, $+|\theta_{\mathrm{t}}|$, which corresponds to a positive transverse momentum for the refracted beam. **(b)** Negative refraction, $-|\theta_{\mathrm{t}}|$, corresponding to a negative transverse momentum for the refracted light.

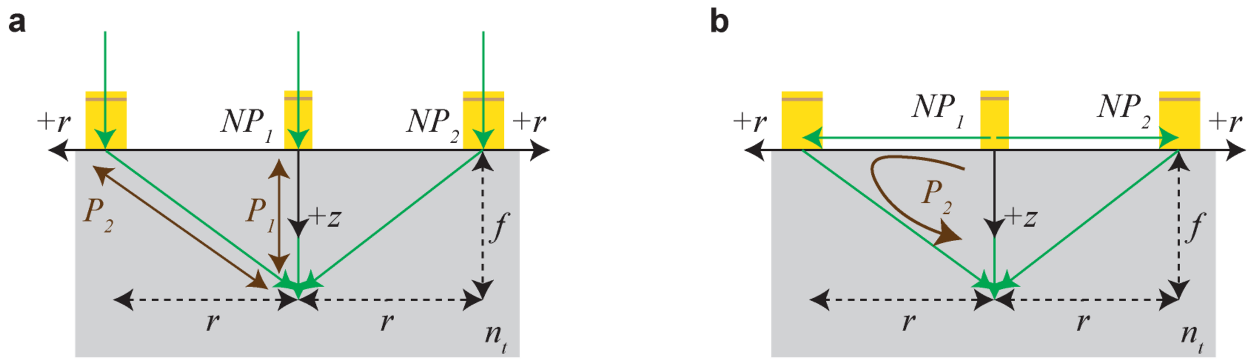

Supplementary Fig. 6: Ray diagram of two metalenses under two different illuminations. *NP₁* corresponds to the nanopillar at the origin and *NP₂* corresponds to the nanopillars placed at a distance of r on either side of *NP₁*. *P₁* and *P₂* represent the paths that the incident light takes once it gets deflected from *NP₁* and *NP₂*, respectively. **(a)** Normal illumination, corresponding to a zero transverse momentum for the illuminating light ($k_{||_i} = 0$). **(b)** Illumination with surface-traveling waves with $|k_{||_i}| = 1.06k_0$.

We can use a similar approach to derive the phase equation for the metalenses. Let us assume that we have a metalens that is designed to focus a normally incident light at a distance of f away from the metalens surface, as shown in Supplementary Fig. 6a. This means that deflected waves from the nanopillars should add constructively at the focal point, necessitating that all these deflected waves should arrive at the focal point with the same phase. In the geometrical optics picture, the phase that each of these waves experiences can be calculated as $\varphi_i = k_0 n_{\mathrm{t}} l$, where l

represents the distance that each wave traverses to arrive at the focal point. In Supplementary Fig. 6a, the phase accumulation equals $k_0 n_\mathrm{t} f$ for the wave deflected from the nanopillar placed at the center (*NP_1*), while it is $k_0 n_\mathrm{t}\sqrt{f^2+r^2}$ for the waves deflected from the other two nanopillars (*NP_2*), placed at distances $|r|$ from the center. To account for this phase difference, *NP_2* should provide a phase of $-k_0 n_\mathrm{t}\sqrt{f^2+r^2}+k_0 n_\mathrm{t} f$ such that all deflected waves arrive at the focal point with equal phases. This basically is the phase equation for a metalens designed to focus a normally incident light[4-6]. If, however, the incident wave is a surface-traveling wave along the $+\hat{\mathbf{r}}$ direction ("outgoing" wave), shown in Supplementary Fig. 6b, the path that the light traverses (and thus the phase accumulation) to reach the focal point becomes different from that of Supplementary Fig. 6a. Specifically, regarding *NP_2*, the phase accumulation along path *P_2* becomes $k_0 n_\mathrm{t}\sqrt{f^2+r^2}+k_{||}r$, where $k_{||}$ is the transverse momentum of the surface-traveling wave. For *NP_1*, however, the phase accumulation is $k_0 n_t f$. To account for this phase difference, *NP_2* should impart a phase of -$k_0 n_\mathrm{t}\sqrt{f^2+r^2}-k_{||}r+k_0 n_\mathrm{t} f$ such that all the waves arrive at the focal point with equal phases. In our designed "outgoing" metalenses, we have used $k_{||}=1.06k_0$. If instead, the surface-traveling wave was traversing along the $-\hat{\mathbf{r}}$ direction ("incoming" wave), we have to use $k_{||}=-1.06k_0$. These two choices will result to the phase equation that we used for the metalenses in the main body of the manuscript [Equation (2)] and are referred to as "outgoing" and "incoming" cases, respectively. Note that if the transverse momentum of the wave approaches 0, we will get the phase equation for a metalens that is designed to focus a normally incident light.

Supplementary Note 5. Light-emitting metalenses

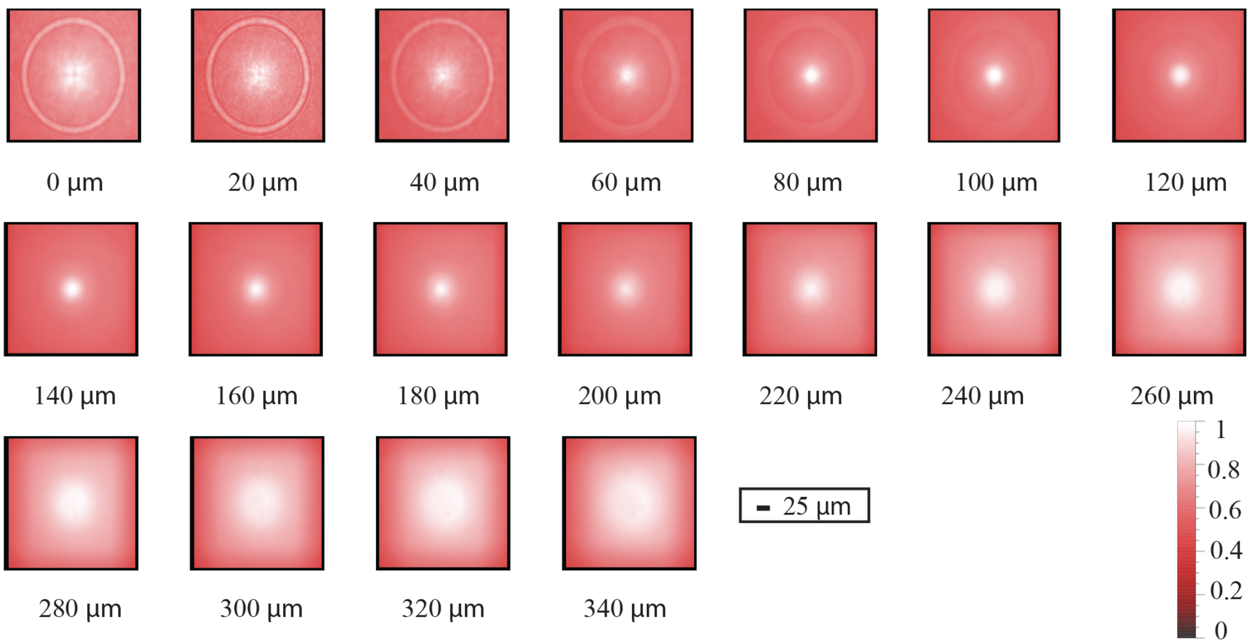

Supplementary Fig. 7: Focusing performance of an "incoming" metalens. The metalens is designed to have a focal length of $f = 150$ µm. The photoluminescence images are shown in increments of 20 µm and show a focused spot between 80-140 µm.

Supplementary Fig. 7 shows the evolution of the produced PL by an "incoming" metalens with a focal length of 150 µm. Supplementary Fig. 8 shows the evolution of the produced PL by an "outgoing" metalens with a focal length of 150 µm. The generated PL shows a ring along with diffractive features up to $z = 180$ µm after which, the ring changes to a central spot. This follows closely from the focal profiles of the metalenses achieved through transmission measurements with structured light, shown in Fig. 3 of the main body of the manuscript, where illumination with higher momenta than the designed momentum led to a ring of light, as opposed to illumination with the deign momentum that created a focal central spot. Supplementary Figs. 8b&c show the beam width and amplitude evolution for the "outgoing" metalenses, calculated using the procedure shown in Figs. 4e&f of the main body of the manuscript. Note that the trends are not as clear as they were for the "incoming" metalenses. Specifically, the point of maximum amplitude is not the same as the point of minimum beam width due to the fact that in these metalenses, the PL profile

changes from a ring to a central spot, hence the differences between the point of maximum amplitude and minimum beam width.

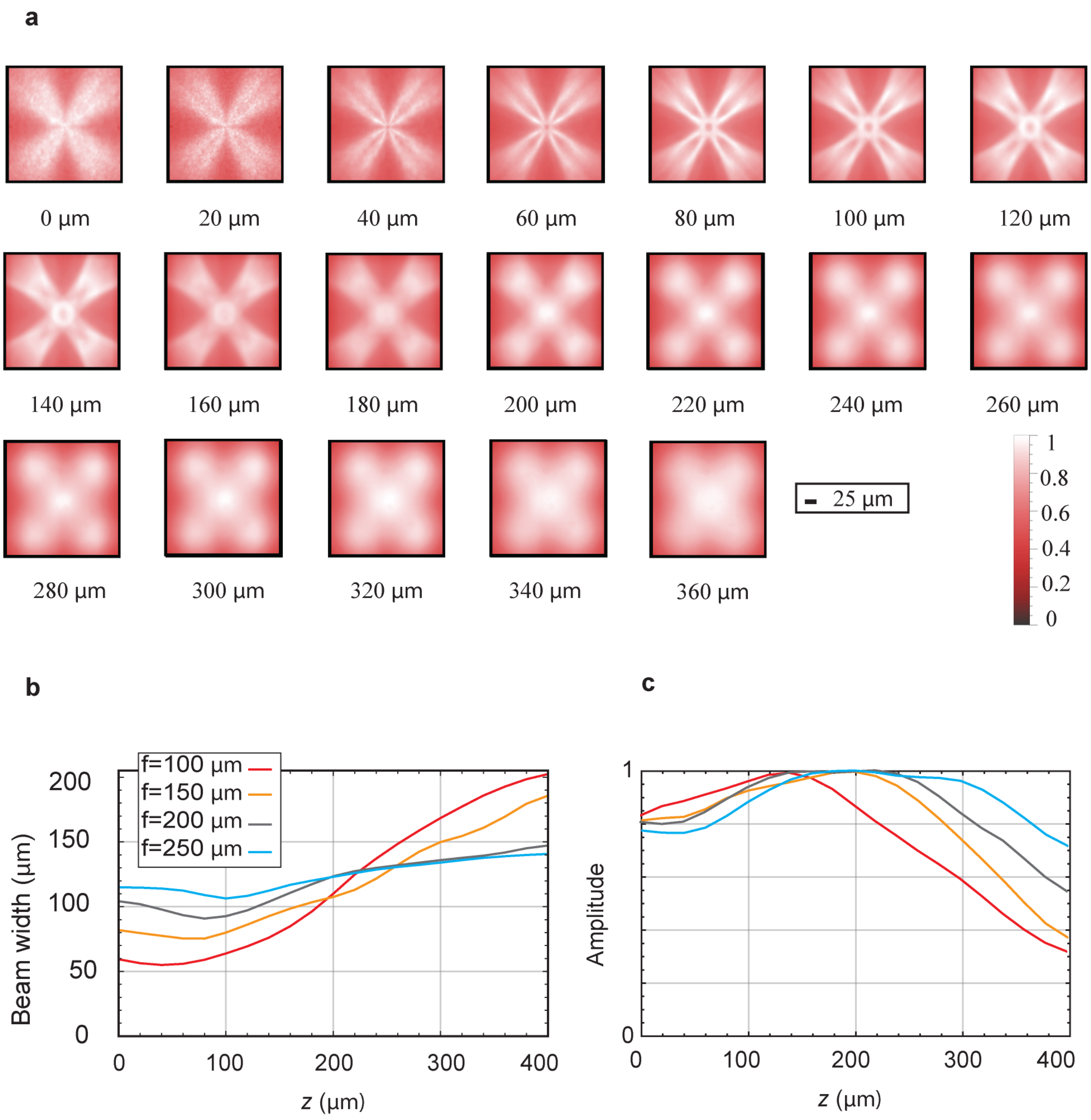

Supplementary Fig. 8: Focusing performance of "outgoing" metalenses. **(a)** Focusing performance of an "outgoing" metalens designed to have a focal length of $f = 150$ µm. The photoluminescence images are shown in increments of 20 µm. **b)** Evolution of the beam width as a function of *z* for "outgoing" metalenses. **c)** Amplitude evolution as a function of *z* for "outgoing" metalenses, normalized to the corresponding maximum value for each metalens.

Supplementary Note 6. Directivity

We define the directivity of our emitting structures as "the ratio of the emission intensity in a given direction from the structure to the emission intensity averaged over all directions"[7]. The average emission intensity is equal to the total power emitted by the structure divided by 4π, which is the whole steradian in the spherical coordinate. Generally, the direction is not specified and therefore, the direction of maximum emission intensity is implied[7]. We can further simplify the directivity definition and say that the directivity of a non-isotropic source is equal to the ratio of its emission intensity in a given direction over that of an isotropic source. Thus, we write the directivity (D) as

$$D = {}^{I}/_{I_0} = {}^{4\pi I}/_{P_{\mathrm{rad}}}, \tag{S4}$$

where, I is the emission intensity of the source and I_0 is the emission intensity averaged over all directions. If the direction is not specified, the direction of maximum emission intensity (maximum directivity) is of interest, expressed as $D_{max} = {}^{I}/_{I_0} = {}^{4\pi I_{\mathrm{max}}}/_{P_{\mathrm{rad}}}$. In this definition, the directivity of an isotropic source is 1. In our measurement setup, NA is limited to 1.3 and so we will not use 4π as the whole steradian space. The maximum emission directivity for the emitting thin film is 1.45 which is not surprising considering that the emission is mainly in the form of a dipole emission (an emission intensity of the form $(\sin(\theta))^2$ has a directivity of 1.5 in the whole steradian space). The directivity for the meta-axicon, however, is 2, an increase of 38%.

Supplementary Note 7. Metalens focusing efficiency in BFP illumination measurements

The 2D focusing efficiency of the metalenses once they are illuminated with a ring of light with a momentum content of $k_0 < |k_{||_{\mathrm{i}}}| < 1.13k_0$ is calculated as follows. We first find the distances from the central spot at which the intensity drops to 10% of the maximum intensity. We calculate

the total number of photons within this region and divide it by the total number of photons in the metalens focal profile. The focusing efficiency values for “incoming” metalenses with focal lengths of 100, 150, 200, and 250 μm are 39%, 49%, 60%, and 70%, respectively. For the “outgoing” metalenses with focal lengths of 100, 150, 200, and 250 μm, the focusing efficiency values are 19%, 23%, 28%, and 36%, respectively. This significantly lower efficiency is expected, since as we saw in Fig. 3b of the main body of the manuscript, we see diffractive features in the images of the focal profiles of the “outgoing” metalenses.

Supplementary Note 8. Metalens-mediated efficiency in PL for “incoming” metalenses

To define the efficiency, we first find the immediate minima on both sides of the peak intensity of the metalens emission (Rayleigh criterion) and calculate the total photon counts within the region between these two points (these points are denoted as “Minima” in Fig. 4c of the main body of the manuscript). We divide this photon count value to the total number of photons within the whole metalens emission intensity, shown as the red curve in Fig. 4c of the main body of the manuscript. Applying this method to 2D PL images in Fig. 4a, we get values of 25%, 31%, 38%, and 45% for the metalenses with focal lengths of 100, 150, 200, and 250 μm, respectively. However, as we can see from Fig. 4c of the main body of the manuscript, the metalens emission lies on top of a background. Therefore, to account for this effect, we subtract the background intensity from the metalens emission intensity, using the approach described in the Methods section of the main body of the manuscript, to find the metalens-mediated component of the emission (see Fig. 4d of the main body of the manuscript). We then calculate the total photon counts contained within the whole region of the curve shown in Fig. 4d of the main body of the manuscript and divide the obtained value to the total number of photons in the metalens emission intensity (red curve in Fig. 4c of the main body of the manuscript). This gives us the metalens-mediated efficiency. For the

2D PL images in Fig. 4a of the main body of the manuscript, the metalens-mediated efficiencies are 4%, 7%, 9%, and 12% for the "incoming" metalenses with focal lengths of 100, 150, 200, and 250 μm, respectively.

Supplementary References:

1. Schuller, J. A. *et al.* Orientation of luminescent excitons in layered nanomaterials. *Nat. Nanotech.* **8**, 271–276 (2013).
2. Taminiau, T. *et al.* Quantifying the magnetic nature of light emission. *Nat. Commun.* **3**, 979 (2012).
3. Yu, N. *et al*. Light propagation with phase discontinuities: generalized laws of reflection and refraction. *Science* **334**, 333–337 (2011).
4. Khorasaninejad, M. *et al*. Metalenses at visible wavelengths: diffraction-limited focusing and subwavelength resolution imaging. *Science* **352**, 1190–1194 (2016).
5. Chen, W. T. *et al*. A broadband achromatic metalens for focusing and imaging in the visible. *Nat. Nanotech*. **13**, 220–226 (2018).
6. Zhang, S. *et al*. High efficiency near diffraction-limited mid-infrared flat lenses based on metasurface reflectarrays. *Opt. Express* **24**, 18024 (2016).
7. Balanis, C. A. *Antenna theory: analysis and design*. (John Wiley, 2005).
